# Rapid Establishment of a Regular Distribution of Adult Tropical *Drosophila* Parasitoids in a Multi-Patch Environment by Patch Defence Behaviour

**DOI:** 10.1371/journal.pone.0020870

**Published:** 2011-07-12

**Authors:** Peter W. de Jong, Lia Hemerik, Gerrit Gort, Jacques J. M. van Alphen

**Affiliations:** 1 Laboratory of Entomology, Wageningen University, Wageningen, The Netherlands; 2 Biometris, Department of Mathematical and Statistical Methods, Wageningen University, Wageningen, The Netherlands; 3 Institute for Biodiversity and Ecosystem Dynamics, University of Amsterdam, Amsterdam, The Netherlands; University of Arkanas, United States of America

## Abstract

Females of the larval parasitoid of *Drosophila*, *Asobara citri*, from sub-Saharan Africa, defend patches with hosts by fighting and chasing conspecific females upon encounter. Females of the closely related, palearctic species *Asobara tabida* do not defend patches and often search simultaneously in the same patch. The effect of patch defence by *A. citri* females on their distribution in a multi-patch environment was investigated, and their distributions were compared with those of *A. tabida*. For both species 20 females were released from two release-points in replicate experiments. Females of *A. citri* quickly reached a regular distribution across 16 patches, with a small variance/mean ratio per patch. Conversely, *A. tabida* females initially showed a clumped distribution, and after gradual dispersion, a more Poisson-like distribution across patches resulted (variance/mean ratio was closer to 1 and higher than for *A. citri*). The dispersion of *A. tabida* was most probably an effect of exploitation: these parasitoids increasingly made shorter visits to already exploited patches. We briefly discuss hypotheses on the adaptive significance of patch defence behaviour or its absence in the light of differences in the natural history of both parasitoid species, notably the spatial distribution of their hosts.

## Introduction

Predators searching a patch for prey remove captured prey items from each patch by consuming them. In contrast, hosts of parasitoids used for oviposition are left in the environment. Hence, they are vulnerable to further attacks by other parasitoids or predators. Because only a limited number of parasitoid eggs per host can develop into adult parasitoids (*e.g.* one for solitary parasitoids), parasitoids are expected to recognize parasitized hosts and to prefer healthy ones for oviposition. Recognition of hosts which are already parasitized, host discrimination, has been found to be widespread, e.g. [1],[2],[3],[4]. In general, parasitoids must compete for a limited number of hosts. When searching together in a patch, conspecific parasitoids are likely to interact with each other, either by directly encountering each other or indirectly through encounters with already parasitized hosts [5]. This may lead to mutual interference, a reduction in the efficiency of parasitism caused by interactions between parasitoids [6]; see [7]. Mutual interference occurs, for example, when a parasitoid encounters conspecifics and stays on the patch longer than it would have without such encounters. Another manifestation of mutual interference is competition by superparasitism (defined as the laying of additional eggs in hosts that have already been parasitized by a conspecific). The parasitoid species *Leptopilina heterotoma* (Thomson) [8],[9],[10] and *Asobara tabida* (Nees) [11] have been found to interfere by superparasitism. When the gain in the number of offspring outweighs the costs involved in searching for unparasitized hosts [1], [12],[13], superparasitism can be adaptive. Superparasitism by a conspecific, however, incurs a cost for the first female that has parasitized a host because her offspring might not survive. Defending the patch against intruding competitors can be an alternative strategy to protect the hosts that have already been parasitized by the defender from superparasitism [14]. Such defence behaviour is another example of mutual interference because it involves the time costs of guarding and patrolling the patch, fighting, and chasing intruders [15]. Both strategies can be adaptive, allowing intruders on the patch and competing by superparasitism or defending the patch against intruding competitors, depending on the circumstances [1]. It is only advantageous to defend a patch under specific conditions. For instance, patches have to be of a defendable size, as has been shown in [16]. Moreover, patch-defence by fighting is only advantageous when patches are found close to each other and travel times are short; the intruder is more likely to leave the patch, and hence the cost of defence will be lower, should alternative opportunities be nearby.

Direct physical interaction between parasitoids exploiting the same patch has not been observed in the field or in the laboratory for the holarctic parasitoid *A. tabida*, although up to eight parasitoids have been observed exploiting one patch without any fighting [5]. For the tropical parasitoid *Asobara citri* (Fisher), spectacular fights have been observed between four females of that simultaneously searched for hosts on a patch in a closed petri dish (P.W. de Jong, J.J.M. van Alphen, pers. obs.). All four parasitoids continued attempting to invade the single patch because they were confined to a petri dish and no alternative patches were present. For each of these four individual parasitoids, the residence time exceeded the time observed for individually searching *A. citri* parasitoids by a factor of four (J.J.M. van Alphen, unpublished data). If parasitoids were offered a number of patches (as in the present study, where 20 wasps were released from two release-points into an arena with 16 patches), one would expect the first *A. citri* female that entered a patch to behave as the owner of the patch and to monopolise and defend it against females arriving later. After being expelled, a female could then move to a nearby patch and defend it if she was the first one to find it. Such a process would quickly result in a regular distribution of parasitoids across patches if the parasitoids do not far outnumber the patches. Thus, the number of actual fights and the loss of foraging time would be minimized. In contrast, females of *A. tabida* that arrive in the same patch after release from local release points should extend their time in a patch to engage in superparasitism. This would result in a clumped distribution of parasitoids until all patches have been exploited. In search of unexploited patches, the wasps would then briefly revisit exploited patches, with a random distribution of wasps across patches as a consequence. This paper describes the results of laboratory experiments designed to test the above predictions.

## Results

### Fighting behaviour

A ‘typical’ fighting event in our experiments was characterised as follows: when an *A. citri* female was foraging on one of the patches, and (an) additional female(s) entered the same patch, a fight would almost always immediately follow. In some cases, it took up to a maximum of 30 seconds before fighting started. Typically, a fighting-‘bout’ lasted only up to 10–20 seconds, after which one of the contestants (usually the ‘intruder’) ran off the patch. The apparent ‘winner’, after pursuing the ‘loser’ across the perimeter of the patch, quickly returned to the patch, and patrolled it by running across it in different directions. This patrolling lasted approximately 10–20 seconds, after which the ‘winner’ resumed foraging if no further intrusion took place. The ‘loser’ could either attempt to re-invade the same patch (after which a new fight ensued between the same contestants), enter a new patch, where, if it was already occupied by another female, a fight would follow between these new contestants, or it could attempt to leave the experimental arena.

To discover whether fighting was correlated with a reduction in the number of parasitoids on a certain patch in the following minute, transitions in numbers of parasitoids from one minute to the next were determined for minutes where fighting did, and did not occur, respectively. The time-unit of one minute was chosen based on the duration of a typical fight as described previously. Only those minutes on each patch with initially two or more females were taken into account, and the five experiments with *A. citri* were pooled. There was a significant association between fighting and a decrease in the number of parasitoids on a patch (χ^2^_2_ = 130.01, *P*≪0.001; Table 1).

**Table 1:** Relation between fighting behaviour and a change in the number of parasitoids on a patch.

	number of parasitoids on a patch		
	increased	did not change	decreased
Fights	14	157	228
No fights	40	371	105

### Spatial distribution

We started with the full model assuming that all four parameters differed between the two parasitoid species. No significant difference, however, was found between *A. citri* and *A. tabida* for parameters *b* and *c*. Hence, the best model to describe the time series of the variance/mean ratio differed only in the limiting value of *a* in eqn. (1) and in *d*, the rate of change between the species. The values (±1 SE) were for *a*_1_ = 0.408±0.081, *a*_2_ = 0.688±0.084, *b* = 0.417±0.093, *c* = −7.90±2.073, *d*_1_ = 0.116±0.011, and *d_2_* = 0.082±0.010. These coefficients resulted in the average models for *A. citri* and *A. tabida* plotted in Fig. 2c.

Thus, the limiting value for the variance/mean ratio (*a*) of *A. citri* was 0.408 and for *A. tabida* 0.688. This suggests a more regular distribution than a Poisson distribution for both parasitoid species, but more extremely so for *A. citri.* A more detailed analysis (see below) revealed that most of the time the distribution of *A. tabida* females across patches was not significantly different from a Poisson distribution, whereas for *A. citri* it was.

In a detailed approach, we took for the five replicates of both species all distributions of parasitoids at five minute intervals (*t* = 5, 10,…., 85 and 90 minutes). We analysed these with the exact variance test for the Poisson distribution using the alternative hypothesis of underdispersion [17]. Thus, if the null hypothesis of being Poisson-distributed was rejected, we could conclude that the distribution of parasitoids was more regular than a Poisson distribution, i.e. the variance was less than the mean. For *A. tabida*, almost none of the distributions were significantly different from a Poisson distribution (Fig. 3): some of them were instead more aggregated at the start of the experiment. For *A. citri*, in contrast, the distribution of parasitoids across patches initially corresponded to a Poisson distribution, after which, within approximately half an hour, most distributions of parasitoids across patches became more regular than the Poisson.

### Simulation model

The results of our calculations show whether patch defence or superparasitism is the best way to compete with conspecifics depending on the travel time between patches and on the density of competitors. Patch defence is only the better strategy when parasitoid densities are relatively low and travel times are short (Fig. 4). When travel times are long the costs of reaching an unexploited patch are high, making it more profitable to stay in the current patch and share it with a competitor. When the density of conspecific competitors is high, the frequency of the arrival of intruders will also be high, making that the time costs of patch defence have to be paid repeatedly. Allowing intruders and competing by superparasitism is then the superior strategy.

## Discussion

**Figure 1:**
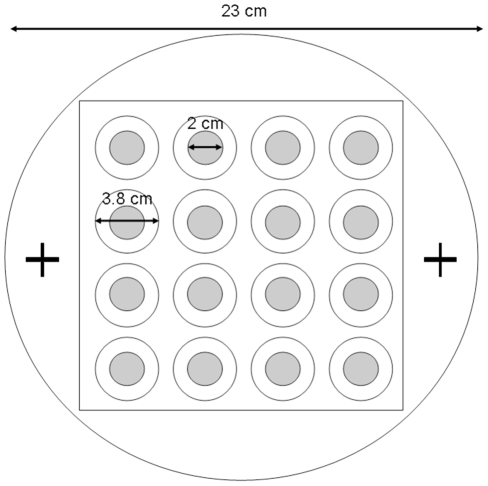
The experimental set-up: the arena consists of a four by four grid of 16 patches. The grey circles represent patches of yeast, containing *Drosophila*-larvae. These are surrounded by an area of agar. Both are level with a circular plastic arena (diameter 23 cm). At each of the “+” marks, 10 parasitoids were introduced into the arena.

**Figure 2:**
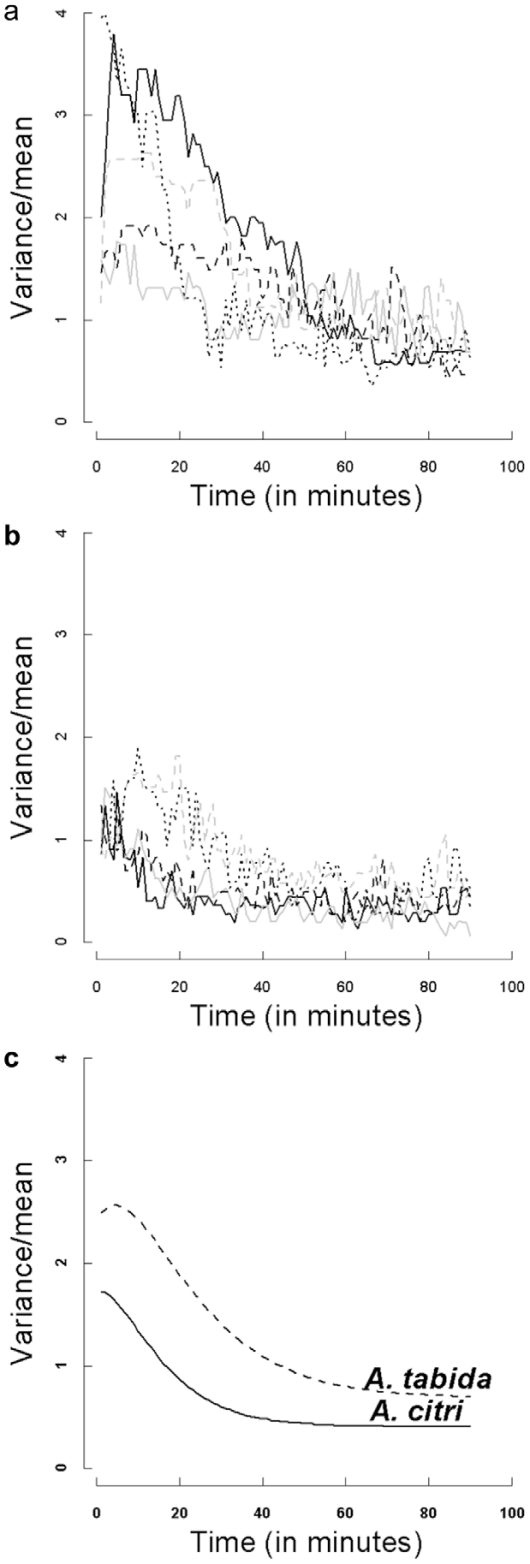
The time series for the variance/mean ratio of the distribution of parasitoids across the 16 patches. (a) the five replicates for *A. tabida*, (b) the five replicates for *A. citri* and (c) the resulting mean transformed Ricker functions from the non-linear mixed model analysis for the two parasitoid species.

The initial distribution, as well as the change in the distributions of adult females across 16 patches clearly differed between *A. citri* and *A. tabida* (compare Fig. 2a with 2b). Whereas within the first half hour of the experiments *A. citri* reached a regular distribution, *A. tabida* initially showed a high degree of clumping that gradually developed into a random, or in some cases, slightly uniform distribution. The rapid formation of a regular distribution for *A. citri* was strongly associated (Table 1) with, and extremely likely to be caused by, the fighting behaviour of the searching females. The initial non-uniform distribution of *A. citri* parasitoids in each experiment can be explained by the way in which the parasitoids were introduced into the arena. They were introduced at two release-points (see Fig. 1): parasitoids were likely to enter the patches closest to the release-site first, and were therefore initially searching together for hosts on a limited number of patches. Those patches at a larger distance from the release-sites initially remained unoccupied. Since each patch was relatively small, the likelihood of parasitoids that were searching together on one patch encountering or detecting each other within a limited amount of time was high. Once this happened, they engaged in a fight, usually resulting in the departure of one of the contestants (Table 1). Since the patches in the arena were relatively close to one another, the defeated wasp was most likely to enter a new patch, which was initially unoccupied. In this case, the defeated wasp usually stayed in the new patch. As more patches gradually became occupied, the chance of a defeated parasitoid entering a patch that was already occupied increased, after which new fights resulted, and so on. This quickly led to a regular distribution of parasitoids across the patches, where most patches contained a single female. Since the experiment was set up in such a way that the total number of parasitoids (20) exceeded the number of patches (16), it was expected that after some time all patches would be occupied by one parasitoid, and occasional fighting would occur by supernumerary parasitoids attempting to invade a patch. This explanation of the process resulting in a regular distribution of *A. citri* across the patches is supported by the observation that in the two experiments where the first fighting occurred later than in the other three experiments, the variance/mean number of parasitoids per patch initially increased to higher levels, and started decreasing later (compare the lightly dashed and the dotted line with the three other lines in Fig. 2b). The reason for the delay in fighting in these two replicates is unknown.

**Figure 3:**
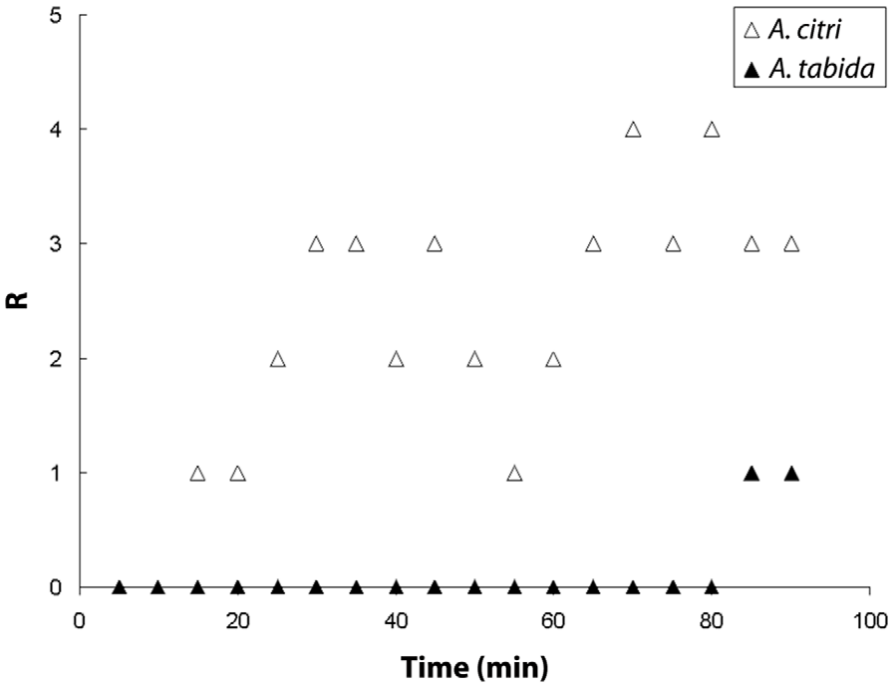
The number of replicates (*R*) with distributions of parasitoids across patches that are more regular than Poisson is determined at 5 minute intervals for all replicate time series (5 for *A. tabida* and 5 for *A. citri*) and plotted against time.

**Figure 4:**
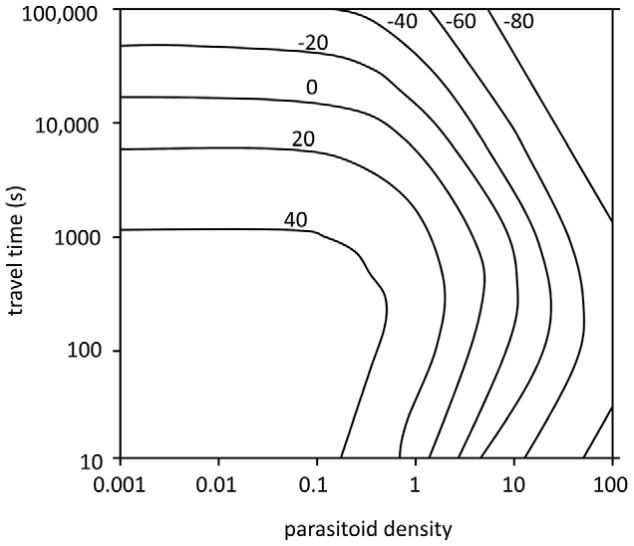
Isoclines of relative fitness by patch defence and superparasitism for an environment with patches with 32 hosts. Ti, the time spent in fighting and chasing an intruder,  = 200 s. On the x-axis the parasitoid density is plotted, and on the y-axis the average travel time between patches in s is plotted. Superparasitism and patch sharing are the better strategy for clines with negative values. Patch defence and fighting is the better strategy for clines with positive values.

*A. tabida* females were released into the arena in the same way as *A. citri* (i.e. two release-points), and hence they also arrived on patches closest to the release sites first. Since no fighting occurred in this species , the variance/mean-ratios could build up rapidly (Fig. 2a) as more and more females entered the same few patches close to the release-sites, and remained there searching for hosts together. Exactly which patch close to the release-sites was invaded first by each parasitoid, and how fast the parasitoids arrived on these patches, was largely a stochastic process. Hence, the initial variation between experiments in variance/mean ratios was expected to be relatively high, and this was indeed found (Fig. 2a). As many as six *A. tabida* females were found searching together on a single patch. In such patches, the limited number (32) of hosts must have been parasitized very rapidly. Females encountering parasitized hosts may decide to start superparasitizing [1], but eventually females will gradually leave such a patch. They should disperse into different directions and enter other patches, which may, or may not already be occupied by (an)other parasitoid(s). Thus, their distribution gradually became less clumped. The distribution can even become slightly uniform if the parasitoids show some variation in residence time, *e.g.* through stochastic variation in encounters of parasitized, vs. unparasitized hosts (e.g. [18]). The total number of “parasite-minutes” per patch was much larger in our present experiments than in earlier work where only one *A. tabida* female was exploiting a patch [19]. This shows that *A. tabida* females in the present experiments were also interfering with each other, probably through superparasitism as described in [11],[12].

The interpretation of the differences observed between the distributions of *A. tabida* and *A. citri* as described above led us to the conclusion that in both species, mutual interference occurs. In *A. citri*, interference involves fighting between adult females leading to their rapid dispersion, whereas *A. tabida* females superparasitize, after which elimination takes place among their larval offspring (note that both *Asobara* species are solitary parasitoids; only one larva per host can develop to become adult). Although it was not studied here, we predict that egg distributions in experiments like these will differ between *A. tabida* and *A. citri*, with less superparasitism in *A. citri*.

*A. citri* females apparently attempt to monopolize a patch by chasing competitors. They spend time fighting and patrolling which they could have otherwise spent searching and parasitizing. A trade-off is therefore expected between time lost in the interactions between the adults, and certainty of maternity through patch monopolization. This trade-off can be influenced by several factors. Patch defence against conspecific competitors is expected to be favoured by selection when hosts develop synchronously. A second factor in favour of patch defence is that the conditions are such that the loser gives up rapidly. These conditions include a high probability of finding a new patch, and low costs of travelling between patches. Third, patches providing a high rate of encounter with suitable hosts are more likely to be economically defendable, and a fourth factor favouring patch defence is that the frequency of intrusions should remain below a certain level.

Considering the natural history of *A.citri* and *A. tabida* may help understand their different behaviour and distribution across patches in light of the conditions mentioned above. *A. citri* parasitizes *Drosophila* larvae in fermenting fruits, mostly figs (J.J.M. van Alphen, pers. obs.), which are often present in large numbers, and for a short period of time, under the canopy of mast-fruiting trees in the tropics. The host density in such fruits is often high [20]. This provides the conditions described above under which patch-defence may be favoured. *A. tabida*, on the other hand, occurs in temperate forests, where it finds its *Drosophila* hosts in fermenting sap fluxes of trees, which may have a large surface area relative to the parasitoid's body size. Compared to the figs in the tropics, the distances between these sap-fluxes are generally large relative to the dispersion-ability of the parasitoids [21]. The encounter rate with *Drosophila* larvae in such sap flows is low [22]. All of these factors are consistent with the conditions making the resource unlikely to be economically defendable. The differences between the temporal and spatial availability of hosts in their natural environment hence are consistent with the difference between *A. tabida* and *A. citri* in likelihood of the evolution of fighting, and therefore may well explain the absence and presence, respectively, of adult fighting behaviour in these species.

## Materials and Methods

### Experimental design

Specimens of *A. citri* collected in the remnants of a rain forest in Ibadan, Nigeria were deposited in the National Centre of biodiversity, Naturalis in Leiden. Some parasitoids were used to set up a laboratory culture at 25°C, on *Drosophila melanogaster* Meigen, strain “Hamburg”. After emergence, parasitoids were stored in groups at 14°C. Successive generations were mixed to prevent inbreeding. Adult parasitoids were fed with honey, and *Drosophila* larvae with yeast (65 g./80 ml. water). For *A. citri* rearing, six groups of parasitoids, each consisting of one male and three females, were allowed to parasitize young second instar *Drosophila* larvae during 24 hours. At 25°C, adult parasitoids emerged approximately 13 days later. *Asobara tabida*, strain “Leiden” had been laboratory reared for many generations as described in [23].

Experiments were carried out with early and late second instar larvae of *D. melanogaster*, strain “WW” (for rearing methods, see [24]), as the host. One day prior to an experiment, 25 female parasitoids that were at the most 10 days old, were “trained” to parasitize hosts as follows: in 25 jars (8.5 cm high, and approximate diameter of 4 cm) a patch of yeast (65 g yeast/80 ml water) was placed on top of an agar layer, and 16 *Drosophila* larvae (24 hours old) were placed on to each patch. Female parasitoids were anaesthetized with carbon dioxide and placed singly into separate jars with these hosts, and the jars were placed in the experimental room, which is described in [25]. The parasitoids were allowed to search and oviposit for 2 hours, and subsequently were collected in one jar with a layer of agar, a drop of honey, and a harmonica-like folded strip of paper for shelter. In the experimental room, they were kept at a 24 H light regime until after the experiment.

A 23 cm diameter plastic disc approximately 1 cm thick, with 16 circular holes (diameter 3.8 cm) arranged in a square (see Fig. 1) served as the experimental “arena”. A layer of agar was applied in the holes up to approximately 1 mm below the rim. A plastic ring (diameter 2 cm, appr. 4 mm thick) was placed in the centre of each of these agar-bottoms. Within these rings 0.4 ml of a 5 g/20 ml yeast suspension was pipetted to form circular patches. When the yeast had dried the rings were carefully removed. At least 30 minutes before the start of an experiment, 32 *Drosophila* larvae, 24 hours old, were introduced on to each patch. Twenty trained parasitoids were selected after being anaesthetized with CO_2_. These were divided into two groups of ten and put into two glass tubes (diameter approximately 0.85 cm, length 8.5 cm) at least 2.5 hours before the start of an experiment. The experiment started by releasing the parasitoids at two opposite sides of the covered arena (indicated by ‘+’ in Fig. 1). The experiment was recorded with a JVC video recorder. At the start and end of the experiment, temperatures were measured (range 20–24°C) and relative humidity was monitored (20–50%).

For one and a half hours, the number of parasitoids on each of the 16 patches was counted every minute from the video-tape recordings. This resulted in a time series of 90 parasitoid distributions. We also scored the occurrence of fighting behaviour during each trial, and as expected, no fights were observed among individuals of *A. tabida*. The experiments were repeated five times per parasitoid species.

### Analysis of data

If all *n* = 16 patches were equally attractive and parasitoids did not influence each other's behaviour, a multinomial distribution of counts of parasitoids across patches was expected, i.e., a parameter vector (*m*; 1/*n*,..,1/*n*). Here, *m* is the total number of parasitoids on the patches, and *n* is the number of patches. If *n* increases and *p*, the probability of being on a certain patch, decreases, the distribution of the count of parasitoids across the patches approaches a Poisson distribution. The multinomial distribution of counts of *m* parasitoids distributed across *n* equally attractive patches has a mean count *m*/*n* per patch with variance *m*(1−(1/*n*))/*n*, and hence the variance/mean ratio will be 1−(1/*n*), *i.e*. 0.94 for 16 patches. An aggregated distribution has a larger variance than the mean, a Poisson distribution (or our multinomial distribution) has a variance that is exactly equal to the mean, and a regular distribution has a variance that is far less than the mean. Hence, the ratio of variance over mean was chosen as the variable of interest.

Each minute, the distribution of parasitoids across the patches was recorded, and the mean and variance of the number of parasitoids per patch were computed. First, the change through time of the variance/mean ratio of the distribution of parasitoids across patches was plotted for each species (Figs. 2a–b). As explained above, the distribution of *A. tabida* over time is not expected to be regular but to converge on a Poisson distribution (with variance/mean≈1). The variance/mean for *A. citri*, however, was expected to drop below zero after a short time depending on the intensity of fighting after the start of the experiment, i.e. the females of this species were expected to distribute themselves much more regularly across the patches. In some replicates, the graphs of the time series showed a variance/mean ratio converging on an asymptotic value after some time (see results, Fig. 2). Therefore, we fitted the following relationship (a ‘Ricker function’ [26]) to the data using R 2.7.0 (R Development Core Team 2008):
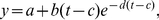
(1)with *y* = variance/mean, *t* = the time in minutes, *a* = limiting value of *y* if the time goes to infinity (∞), *b* scales the speed of decline, *c* is a delay factor (if positive) and *d* is a measure of the rate of decline of *y*.

In our experiment, the unit was an arena with 16 patches containing 20 simultaneously released parasitoids. As the variance/mean ratios were repeatedly measured (every minute) on each experimental unit, an independence of observations cannot be assumed. To account for possible correlations between observations from the same experimental unit, we propose a random coefficients non-linear model as described in eqn. (1).

Each experimental unit has its own non-linear model:
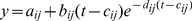
(2)with *i* representing the parasitoid species (*i* = 1: *A. citri*, *i* = 2: *A. tabida*), and *j* the index for replication within each species (*j* = 1, …, 5). We assume that parameters *a_ij_* form a random sample from normal distributions, 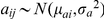, with a mean that may depend on species, and we make similar assumptions for parameters *b_ij_*, *c_ij_*, and *d_ij_*. The vector (a*_ij_*, *b_ij_*, *c_ij_*, *d_ij_*) has variance-covariance matrix Σ, with diagonal 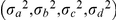 and non-specified covariances.

For each of the parameters, e.g. *a_ij_*, we used likelihood ratio tests to determine whether the means *μ_a_*_1_ and *μ_a_*_2_ for the two species were equal. If no significant difference was found, we simplified the model, and assumed both means were equal, e.g. 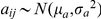. We used the software package **nlme** [27] to fit equation (2) as a non-linear mixed effect model to the full data set [27] allowing random effects for all four parameters *a*, *b*, *c* and *d* as explained above.

Because we had five replicates of the full distribution of parasitoids of each species across the patches for each moment in time, we could determine whether the average distributions of *A. tabida* and *A. citri* differed from a Poisson distribution at five minute intervals starting from *t* = 5 minutes until the end of the observational period (*t* = 90 minutes). Comparisons were made using the exact variance test for the Poisson distribution, with as an alternative hypothesis of underdispersion (i.e. more regular than Poisson, [17]).

We scored fights between *A. citri* females each minute; fighting between different pairs of parasitoids were considered different fights, successive fighting bouts between the same females within one minute were treated as one fight. Any changes in the number of *A. citri* females on each patch were correlated with the occurrence of fighting behaviour on that patch using a chi-squared test.

### Simulation model

To investigate under which conditions the spatial distribution of host patches favours the evolution of patch defence and when competition by superparasitism is favoured, we calculated the fitness of parasitoids with different strategies under various conditions with a simulation model. In this analysis, wasps are the first to enter a patch with probability *p* and arrive in an already occupied patch with probability 1–*p*. The probability of arriving first decreases with parasitoid density in the habitat. In the simulations for calculating the fitness of parasitoids that competed by superparasitism, we used the Visser et al. (1992) ESS model [9] for superparasitism to calculate patch times for wasps that play the superparasitism game. For wasps that defend patches, we used the same model [9] to calculate patch times for wasps searching a patch alone. These wasps do not superparasitise and do not spend time defending patches. We then increased these patch times with the time costs of fighting and chasing as measured in our experiments with *A.citri* (see the first part of the results section below). The latter was done because we have no deductive model for predicting the time cost of patch defence. This is a reasonable approach because these time costs are not under control of the defending female. We varied travel times to obtain different threshold rates for patch leaving, using Charnov's marginal value theoremto study how travel time affects the competitive strategy favoured in a particular habitat. We varied *p* by varying the wasp density in the habitat. The simulated wasps foraged during 100 hours in an environment containing 100 patches. We calculated fitness as the number of realised offspring [9]. For each combination of travel time and wasp density we then calculated the difference in offspring numbers between wasps following a superparasitism strategy and wasps defending patches against intruders.
